# Using random-forest multiple imputation to address bias of self-reported anthropometric measures, hypertension and hypercholesterolemia in the Belgian health interview survey

**DOI:** 10.1186/s12874-023-01892-x

**Published:** 2023-03-25

**Authors:** Ingrid Pelgrims, Brecht Devleesschauwer, Stefanie Vandevijvere, Eva M. De Clercq, Stijn Vansteelandt, Vanessa Gorasso, Johan Van der Heyden

**Affiliations:** 1 Service Risk and Health Impact Assessment, Sciensano, Rue Juliette Wytsman 14, 1050 Brussels, Belgium; 2 Applied Mathematics, Computer Science and Statistics, Ghent University, Krijgslaan 281, S9, BE-9000 Ghent, Belgium; 3 Department of Epidemiology and Public Health, Sciensano, Rue Juliette Wytsman 14, 1050 Brussels, Belgium; 4 Department of Translational Physiology, Infectiology and Public Health, Ghent University, Salisburylaan 133, Hoogbouw, B-9820 Merelbeke, Belgium; 5 Department of Public Health and Primary Care, Ghent University, Corneel Heymanslaan 10, 9000 Ghent, Belgium

**Keywords:** Measurement error, Multiple imputation, Health interview survey, Health examination survey, Obesity, Hypertension, Hypercholesterolemia

## Abstract

**Background:**

In many countries, the prevalence of non-communicable diseases risk factors is commonly assessed through self-reported information from health interview surveys. It has been shown, however, that self-reported instead of objective data lead to an underestimation of the prevalence of obesity, hypertension and hypercholesterolemia. This study aimed to assess the agreement between self-reported and measured height, weight, hypertension and hypercholesterolemia and to identify an adequate approach for valid measurement error correction.

**Methods:**

Nine thousand four hundred thirty-nine participants of the 2018 Belgian health interview survey (BHIS) older than 18 years, of which 1184 participated in the 2018 Belgian health examination survey (BELHES), were included in the analysis. Regression calibration was compared with multiple imputation by chained equations based on parametric and non-parametric techniques.

**Results:**

This study confirmed the underestimation of risk factor prevalence based on self-reported data. With both regression calibration and multiple imputation, adjusted estimation of these variables in the BHIS allowed to generate national prevalence estimates that were closer to their BELHES clinical counterparts. For overweight, obesity and hypertension, all methods provided smaller standard errors than those obtained with clinical data. However, for hypercholesterolemia, for which the regression model’s accuracy was poor, multiple imputation was the only approach which provided smaller standard errors than those based on clinical data.

**Conclusions:**

The random-forest multiple imputation proves to be the method of choice to correct the bias related to self-reported data in the BHIS. This method is particularly useful to enable improved secondary analysis of self-reported data by using information included in the BELHES. Whenever feasible, combined information from HIS and objective measurements should be used in risk factor monitoring.

**Supplementary Information:**

The online version contains supplementary material available at 10.1186/s12874-023-01892-x.

## Background

Worldwide, 63% of deaths are caused by non-communicable diseases (NCDs). A high proportion of NCDs are preventable by addressing their main physiological risk factors, such as high blood pressure, obesity and hypercholesterolemia [1]. Accurate data on the prevalence of these risk factors is therefore essential to build evidence-based prevention programs and policies [2]. In many countries, the prevalence of NCDs risk factors is commonly assessed through self-reported information from health interview surveys. It has been shown, however, that relying on self-reported data lead to an underestimation of the prevalence of overweight and obesity [3–6], hypertension [7–10] and hypercholesterolemia [11–16]. Social desirability or lack of knowledge may explain the overall validity problem. In addition to biased prevalence estimates, the measurement error related to self-reported data can also bias the estimated association between exposure and disease [17, 18]. In particular, exposure-disease associations are often attenuated when based on self-reported exposures [19, 20]. Although a large body of literature already exists on methods to obtain more accurate surveillance data by correcting for measurement error related to self-reported data, few epidemiologic studies use them in practice [21, 22].

Promising methods to correct for measurement error are based on validation sampling, whereby an accurate measure is collected for a random subset of units of a large study, which are sampled according to a pre-specified design [20]. Regression calibration is the most commonly applied method to correct for measurement error related to self-reported data [22]. This method uses the information that maps objective clinical values to self-reported values by fitting a regression model for the clinical values [23]. All self-reported values in the regression model of interest are then replaced by the predicted clinical values. This method is popular because of its simplicity, but known to leave residual bias; standard error calculations are moreover complicated by the fact that they must acknowledge uncertainty in the predicted clinical values [24, 25]. For the correction of BMI, in particular, it has been shown that the characteristic pattern of error associated with self-reported BMI is practically impossible to correct by the use of linear regression models [26]. Although regression calibration may be an adequate tool to produce valid prevalence estimates of obesity, hypertension or hypercholesterolemia in a population, this method is limited if researchers are interested in using these adjusted factors as predictor variables for modelling disease. A previous study showed that both self-reported and corrected BMI from regression model resulted in biased estimates of association [27].

Some of these concerns can be overcome via multiple imputation, a well-established method of handling missing data that is also useful in dealing with exposure measurement error, known as MIME (Multiple Imputation for Measurement Error) [23, 25, 28–30]. It fills in the missing data with plausible clinical values, which are randomly drawn from a distribution of predicted values. This process is repeated multiple times, the final analysis is carried out on each filled-in dataset, and the results are pooled using Rubin's rules [31]. Multiple imputation has the advantage to produce unbiased estimates and valid inference for data that are correctly modelled and obey missingness at random (MAR) [32]. In the case of measurement error where a validation study is available, MAR is satisfied because the validation subset is chosen completely at random so that people with versus without error-prone measurements are comparable [18]. Within the multiple imputation by chained equations algorithm (MICE), imputed values for one variable are drawn from a predictive model based on all other variables. The process then cycles through each variable imputation until convergence. A misspecified imputation model may however give biased estimates and invalid inferences. Attention is therefore shifting towards the use of a machine-learning-based imputation technique, the random-forest algorithm [32–35]. Random-forest is an algorithm which combines the output of multiple decision trees to solve classification or regression problems. Besides their ability to handle data with complex interaction or non-linearity, those techniques do not require to specify an imputation model and allow the inclusion of a large number of predictors [25, 34, 36]. Furthermore, It has been demonstrated that, in complex settings, those methods produce more plausible imputations and more reliable inferences than standard regression imputation techniques [34, 37].

In Belgium, the prevalence of physiological risk factors is assessed on a regular basis via self-reported information from the Belgian Health Interview surveys (BHIS 1997–2018) [38]. Additionally, small-scale surveys such as the Food consumption surveys (FSC 2004, 2014) [39], the Belgian Health examination survey (BELHES 2018) [40] provide objective measurements, albeit on a smaller subset of the population. Since the BELHES 2018 was conducted on a sub-sample of the BHIS 2018, this joint dataset provides a unique opportunity to assess the validity of self-reported information on physiological risk factors.

The objective of this study is threefold: 1) to assess the agreement between self-reported and measured information on height, weight, hypertension and hypercholesterolemia in Belgian adults and examine how the use of self-reported data impacts the estimates of the prevalence of those risk factors, 2) to identify an adequate approach for valid measurement error correction by comparing regression calibration with MICE based on parametric and non-parametric techniques and 3) to enrich the BHIS 2018 dataset with imputed clinical values for height, weight hypertension and hypercholesterolemia allowing researchers to improve their analysis of self-reported data in the BHIS 2018.

## Methods

### Study area, study population and data

The study area is the entire Belgian territory with a population of 11.4 million inhabitants.

The study sample consists of 9439 participants of the BHIS 2018 older than 18 years including a subset of 1184 participants who additionally participated to the BELHES 2018.

The BHIS is a national cross-sectional population survey carried out every five years by Sciensano, the Belgian institute of health, in partnership with Statbel, the Belgian statistical office. Data are collected through a stratified multistage, clustered sampling design (approximately 10,000 participants) and weighting procedures are applied to obtain results which are as representative as possible of the Belgian population. Data are obtained on socio-economic status, physical and mental health, lifestyle and use of health care [38, 40, 41].

In the BELHES, objective health information was collected among a random subsample of the BHIS participants. In the BELHES, objective health information was collected among a subsample of the BHIS participants. A random subsample of eligible BHIS participants (at least 18 years and having participated in the BHIS themselves) was invited to participate in the BELHES. Recruitment from among this subsample continued until a predefined number of participants was reached. Finally, 1184 individuals participated in the BELHES. The BELHES followed as much as possible the methodological guidelines provided in the framework of the European Health Examination Survey initiative [42].

Data were collected at the participant’s home by trained nurses. The BELHES included a short additional questionnaire, a physical examination and the collection of a blood sample. The physical examination consisted of the measurement of height, weight, waist circumference, blood pressure and for people aged 50 years and above a handgrip measurement. Laboratory blood analyses included the measurement of total and HDL serum cholesterol. Details on the data collection are available in the BELHES publication [40].

### Statistical analyses

In a first step, the merged BHIS/BELHES 2018 database was used (*n* = 1184) to assess the validity of the self-reported data related to height, weight, overweight, obesity, hypertension and hypercholesterolemia. The definitions of the variables for measured and self-reported data are given in Table 1. The difference in the prevalence of self-reported versus measured risk factors was assessed using the McNemar test for paired data. Confusion matrix and Kappa coefficients were used to assess the agreement between self-reported and measured hypertension, hypercholesterolemia and WHO BMI categories. Bland & Altman plots and Intra Class Correlation coefficients (ICC) were used to assess agreement between self-reported and measured height, weight and BMI [43]. The mean difference was assessed using the paired-t test. In Bland & Altman plots,

**Table 1:** Definition of indicators from the Belgian health interview survey (BHIS) and the Belgian health examination survey (BELHES)

Indicator	Variable definition in the BHIS (SR data)	Variable definition in the BELHES (Measured data)
BMI	SR weight (kg)/(SR height (m)) ^2^	Measured weight (kg)/(Measured height (m)) ^2^
Weight	SR weight (kg)	Measured weight (kg)
Height	SR height (cm)	Measured height (cm)
Overweight	BMI, based on SR data ≥ 25 kg/m^2^	BMI, based on measured data ≥ 25 kg/m^2^
Obesity	BMI, based on SR data ≥ 30 kg/m^2^	BMI, based on measured data ≥ 30 kg/m^2^
Hypertension	Has answered “Yes” to question “Did you suffer from hypertension in the last 12 months?”	Systolic blood pressure ≥ 140 mmHg or diastolic blood pressure > 90 mmHg or medication use for hypertension
Hypercholesterolemia	Has answered “Yes” to question “Did you suffer from high cholesterol in the last 12 months?”	Total serum cholesterol > 190 mg/dl

*SR* Self-reported

Horizontal lines are drawn at the mean difference, and at the limits of agreement, defined as the mean difference plus and minus 1.96 times the standard deviation of the differences. Sensitivity, specificity, positive predictive value (PPV) and negative predictive value (NPV) were calculated for each risk factor (overweight, obesity, hypertension and hypercholesterolemia). The parameters of accuracy were stratified by age, gender and education level.

In a second step, different methods to correct for measurement error related to the self-reported risk factors were applied to the complete BHIS 2018 dataset (*n* = 9439). Prevalence of overweight, obesity, hypertension and hypercholesterolemia were compared using regression calibration, MICE based on parametric and non-parametric techniques.

To correct for measurement error with regression calibration, a regression model was fitted to predict the measured health condition based on the self-reported health condition, age, sex and highest educational level in the household of the participant. Interaction terms between the self-reported health condition and covariates were added in the model when they significantly improved the accuracy of the model at the 5% significance level (Wald test). The sample was separated in a training (70%) and a test dataset (30%) to calculate the predictive power of each regression model, assessed by the means of the R^2^ for the linear regression model and by the means of the Area Under the Curve (AUC) for the logistic regression models. The self-reported values were then replaced by the predicted values obtained from the linear regression (for height and weight) and logistic regression (for hypertension and hypercholesterolemia) and the corrected BMI value was finally calculated based on the predicted values for height and weight.

To correct for measurement error with multiple imputation, the measurement error related to self-reported data was treated as a missing data problem. This means that all BHIS participants who were not included in the BELHES were considered with missing values for the measured height, weight, hypertension, and hypercholesterolemia. The missing data pattern of the variables of interest of the merged BELHES/BHIS 2018 dataset can be visualized in Additional file 1.

Unlike the regression calibration, the model was used to multiply impute the measured values for the BHIS. A MICE algorithm [25] was used to multiply impute the missing values of the measured height, weight, hypertension and hypercholesterolemia for every BHIS 2018 participant. Two multiple imputation techniques were compared: a parametric approach based on the predictive mean matching method and logistic regressions and a non-parametric random-forest approach. The imputation model included the same variables that were used for the regression calibration: main effects of age, sex, education level and the self-reported health condition [21]. In addition, variables related to the sample design, such as household size and province were also taken into account in the imputation model.

The number of imputations was limited to 10 to create a small number of completed datasets in public-use data for the convenience of analysts. The relative efficiency was computed to assess if an additional number of completed datasets could reduce the SE of the parameters. The relative efficiency above 99% indicated that 10 completed dataset was sufficient. In particular, using infinitely many imputations would only reduce the variance of the estimators by 1%. The number of iterations of the MICE algorithm was 100. For the random-forest based imputation, the defined number of trees was set to 100. All missing values of the covariates included in the imputation models were imputed in the same process. The convergence of the algorithm was assessed by plotting the mean and standard deviation of the synthetic values against the iteration number for the imputed BHIS data.

Risk factor prevalence estimates were calculated in each completed dataset and results of the multiple analysis were pooled using the standard Rubin rules [31]. Corrected prevalence estimates were obtained by taking the survey weights relative to the sample design into account. Standard errors of the prevalence estimates were obtained as the square root of the total variance (taking into account the within and between imputation variance and a correction factor for using 10 imputations).

Sensitivity analyses were carried out using a wider set of self-reported variables that could potentially be related to the measured health conditions of interest such as socio-economic, lifestyle and health condition variables (the list of the wide set of variables is available in Additional file 2). Missing data of all variables included in the imputation model were imputed in the same process. Finally, the ability of the imputation model to predict valid national estimates for the previous BHIS waves was assessed by applying the random-forest multiple imputation model to the complete BHIS2008/2013/2018 dataset and adding the year in the imputation model.

All statistical analysis were performed by taking into account the survey weights, strata and clusters relative to the sample design. For multiple imputation, the variables used in the weighting procedure (province, number of persons by household, age and sex) were included in the imputation model. All analyses were fit and evaluated using the statistical software R [44], version 4.2.1 (R Development Core Team, 2006) and the “MICE” package [45].

## Results

### Data description

Summary statistics of all considered variables are displayed in Additional file 3.

### Validity of self-reported height, weight, bmi, hypertension and hypercholesterolemia

#### Height, weight and BMI

There was a high agreement between self-reported and measured data for height and weight (ICC for height: 0.95; 95% CI [0.94;0.95], ICC for weight: 0.96; 95% CI [0.95;0.97]). On average, people tended however to overestimate their height by 1.05 (95% CI [-0.83;1.29]) cm and underestimate their weight by 1.50 kg (95% CI [-1.81;-1.20]) (Table 2). This trend was more pronounced for women and older people. While the bias for height was higher among low educated people, the bias for weight was higher among high educated people. Bland–Altman plots illustrating the agreement between self-reported and measured height and weight stratified by age, gender and education level are available in Additional files 4, 5, 6, 7, 8 and 9.

**Table 2:** Estimates of the Bland–Altman plots for analysis of agreement between self-reported and measured height, weight and Body Mass Index (BMI) stratified by age

		**Weighted mean difference**	**LLOA**	**ULOA**
BMI (kg/m^2^) [95% CI]	**Whole population**	-0.87 [-1;-0.74]	-4.31 [-4.48;-4.14]	2.63 [2.45;2.80]
	**Age category**			
	18/24	-0.59 [-0.95;-0.23]	-3.41[-4.03;-2.79]	2.23 [1.61;2.85]
	25/44	-0.67 [-0.83;-0.52]	-3.74 [-4.0;-3.48]	2.40 [2.13;2.66]
	45/64	-0.74 [-0.92;-0.55]	-4.72 [-5.04;-4.4]	3.25 [2.93;3.58]
	> 65	-1.35 [-1.54;-1.16]	-4.36 [-4.68;-4.04]	1.64 [1.32;1.97]
	**Gender**			
	Men	-0.61 [-.077;-0.45]	-4.24 [-4.50;-3.98]	2.94 [2.68;3.02]
	Women	-1.12 [-1.30;-0.94]	-4.34 [-4.57;-4.11]	2.31 [2.08; 2.34]
	**Education level**			
	No diploma/ prim	-1.27 [-2.11;-0.42]	-7.34 [-8.80;-5.86]	4.80 [3.34;6.25]
	Lower secondary	-0.93 [-1.27;-0.58]	-4.55 [-5.14;-3.95]	2.69 [2.10;3.29]
	Higher secondary	-0.87 [-1.09;-0.65]	-5 [-5.40;-4.65]	3.27 [2.87;3.64]
	Higher	-0.7 [-0.86-;-0.66]	-3.31 [-3.48;-3.13]	1.78 [1.60;1.95]
Height (cm) [95% CI]	**Whole population**	1.05 [0.83;1.29]	-4.7 [-4.99;-4.41]	6.71 [6.42;7]
	**Age category**			
	18/24	0.38 [-0.27;1]	-4.75 [-5.88;-3.63]	5.51 [4.39;6.64]
	25/44	0.35 [0.10;0.60]	-4.69 [-5.12;-4.25]	5.40 [4.97;5.84]
	45/64	0.78 [0.53;1.03]	-4.57 [-5.0;-4.14]	6.14 [5.71;6.58]
	> 65	2.56 [2.16;2.96]	-3.76 [-4.45;-3.03]	8.89 [8.20;9.58]
	**Gender**			
	Men	0.69 [0.43;0.94]	-4.37[-4.74;-3.99]	5.99 [5.62;6.37]
	Women	1.41 [1.06;1.75]	-3.74 [-3.48;-4.0]	7.31 [6.88;7.74]
	**Education level**			
	No diploma/ prim	2.32 [0.80;3.83]	-8.55 [-11.55;-5.94]	13.19 [10.6;15.79]
	Lower secondary	1.35 [1.82;1.88]	-4.13 [-5.03;-3.23]	6.84 [5.93;7.75]
	Higher secondary	1.30 [0.99;2.96]	-4.49 [-5.01;-3.97]	7.10 [6.57;7.63]
	Higher	0.64 [0.43;0.82]	-4.01 [-4.36;-3.72]	5.32 [4.99;5.64]
Weight (kg) [95% CI]	**Whole population**	-1.50 [-1.81;-1.20]	-9.75 [-10.2;-10.3]	6.89 [6.47;7.31]
	**Age category**			
	18/24	-1.42 [-2.46;-0.37]	-9.64 [-11.44;-7.8]	6.80 [5;8.60]
	25/44	-1.62 [-2;-1.23]	-9.20 [-9.54;-8.54]	5.95 [5.30;6.61]
	45/64	-1.33 [-1.83;-0.85]	-11.4 [-12.2;-10.6]	8.77 [7.96;9.59]
	> 65	-1.76 [-1.71;-1.01]	-6.87 [-7.47;-6.27]	4.15 [3.15;4.74]
	**Gender**			
	Men	-1.26 [-1.72;-0.81]	-11.1 [-11.9;-10.4]	8.66 [7.93;9.38]
	Women	-1.73 [-2.14;-1.32]	-8.20 [-8.65;-7.74]	4.95 [4.45;5.41]
	**Education level**			
	No diploma/ prim	-0.89 [-2.32;0.53]	-11.13 [-13.59;-8.68]	9.34 [6.88;11.79]
	Lower secondary	-1.26 [-2.04;-0.48]	-9.44 [-10.74;-8.06]	6.88 [5.53;8.22]
	Higher secondary	-1.29 [-1.87;-1.72]	-12.1 [-13.15;-11.16]	9.55 [8.57;10.54]
	Higher	-1.62 [-1.88;-1.37]	-7.91 [-8.35;-7.48]	4.66 [4.23;5.1]

*LLOA* Lower limits of agreement, *ULOA* Upper limits of agreement (mean difference ± 2 standard deviations)

**Fig. 1:**
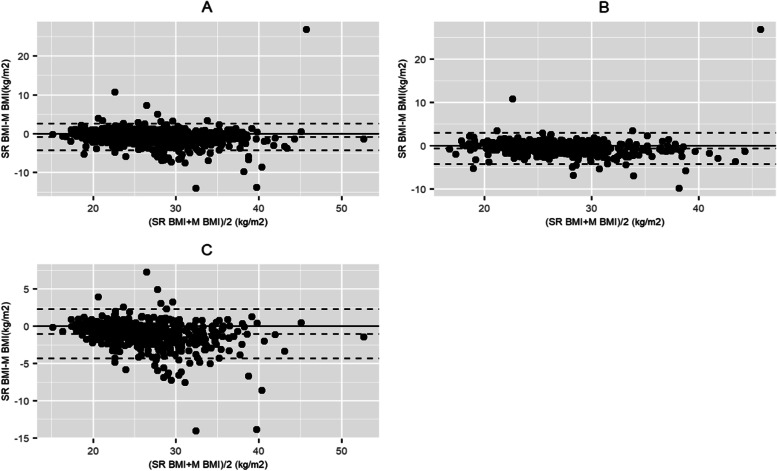
Bland–Altman plot for analysis of agreement between self-reported and measured Body Mass Index (BMI), for the whole population and by gender. **A** Whole study population, **B**: Men, **C**: Women. The solid line represents the mean difference. The dashed lines represent the upper and lower limits of agreement (mean difference ± 2 standard deviations)

The agreement between self-reported and measured BMI was slightly lower than for height and weight (ICC: 0.92; 95% CI [0.86;0.95]). Figure 1 illustrates the agreement between self-reported and measured BMI separately for men and women. The mean bias for the whole population was close to zero (-0.84 kg/m^2^) indicating a very good agreement at the population level.

The lower limit of agreement (LLOA) and upper limit of agreement (ULOA) revealed however a wider variability at the individual level (Table 2). The plots in Fig. 1 show that people with overweight (> 25 kg/m^2^) were more likely to underestimate their BMI. The stratified analysis indicated a more pronounced misreporting bias among women, older and low educated people (Table 2). Bland–Altman plots for BMI stratified by age and education level are available in Additional files 10 and 11.

The agreement between self-reported and measured BMI categories was high with 82% of the participants correctly classified (Kappa: 73%). The prevalence of obesity (BMI > 30 kg/m^2^) was however significantly underestimated when based on self-reported body weight and height (Table 3). Using self-reported BMI allowed us to detect only 78% of BHIS participants with overweight and 69% of BHIS participants with obesity (Table 3).

**Table 3:** Prevalence estimates of overweight, obesity, hypertension and hypercholesterolemia using self-reported data (BHIS 2018) and measured data (BHES 2018). Sensitivity, specificity, positive predictive value (PPV) and negative predictive value (NPV) using self-reported data

	Overweight (%)			Obesity (%)		
	Total	Men	Women	Total	Men	Women
Prevalence BELHES [95% CI]	34 [31-38]	39 [34-45]	29 [25-34]	22 [19-25]	20 [16-24]	22 [18-27]
Prevalence BHIS [95%IC]	34 [30-37]	39 [34-44]	29 [24-33]	15 [13-18]	14 [11-18]	16 [13-21]
Sensitivity	78	80	73	69	69	65
Specificity	88	86	89	99	99	99
PPV	78	80	75	94	95	93
NPV	88	86	85	92	92	91
	Hypertension (%)			Hypercholesterolemia (%)		
	Total	Men	Women	Total	Men	Women
Prevalence BELHES [95% CI]	33 [29-36]	33 [28-38]	33 [28-38]	47 [43- 51]	46 [40-51]	48 [43-53]
Prevalence BHIS [95%IC]	16 [13-19]	15 [12-19]	17 [13-21]	21 [18- 24]	18 [14-2]	22 [17-27]
Sensitivity	45	41	51	22	41	18
Specificity	99	97	98	83	78	87
PPV	90	88	91	56	45	66
NPV	79	74	83	52	54	51

^*^CI Confidence interval. Significant underestimation of the prevalence estimates for obesity, hypertension and hypercholesterolemia in the BHIS (*P* < 0.001*)*

By contrast, the high specificity rates indicates that self-reported BMI is a reliable indicator to rule out the existence of overweight and obesity. Prevalence estimates stratified by age and education level are available in Additional files 12 and 13.

#### Hypertension and hypercholesterolemia

**Fig. 2:**
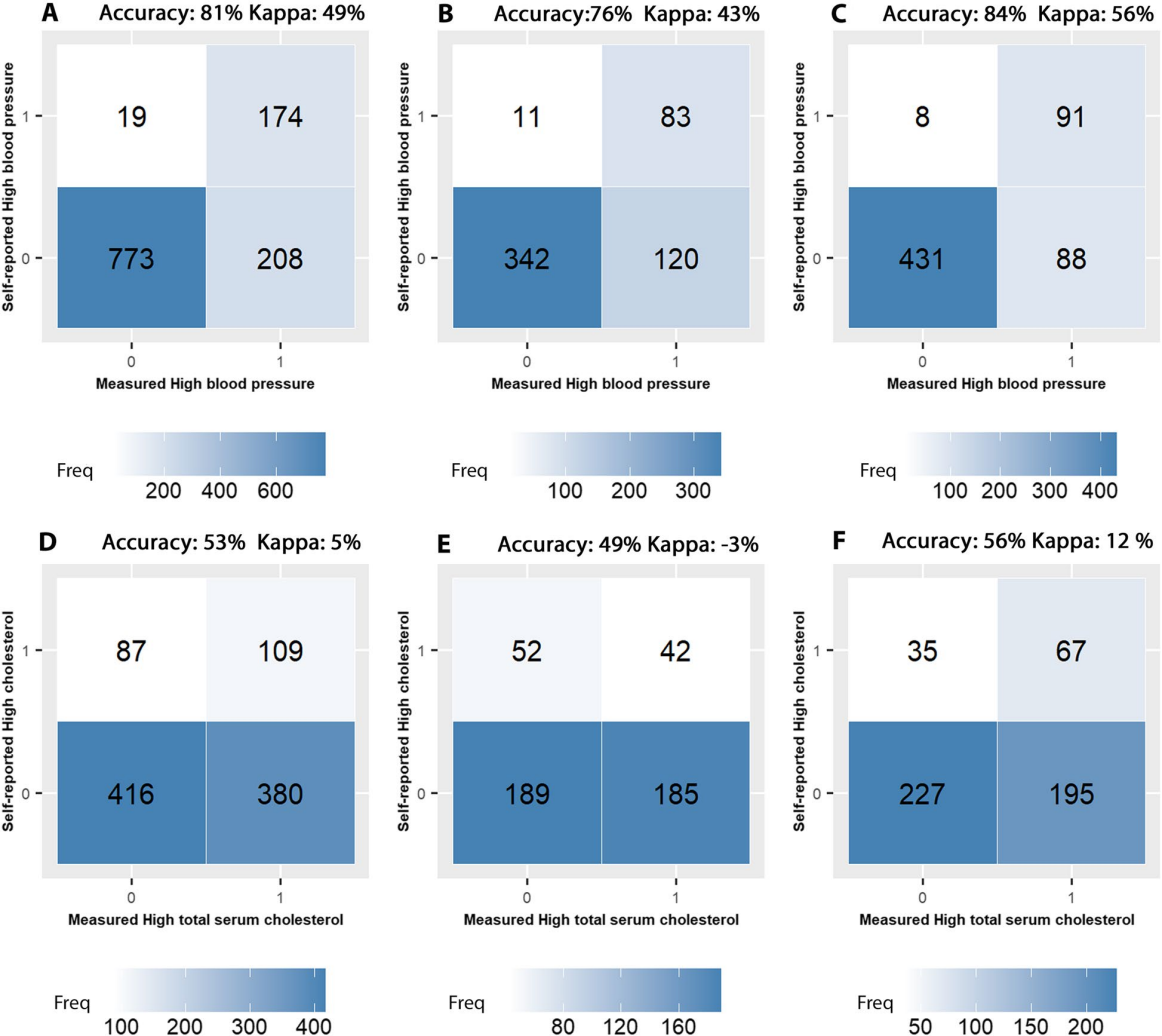
Confusion matrix comparing self-reported and measured high blood pressure and hypercholesterolemia, for the whole population and by gender. **A **and** D**: Whole study population, **B **and** E**: Man, **C **and** F**: Women

There was a moderate agreement between self-reported and measured hypertension, with a Kappa coefficient of 0.49 (Fig. 2). The agreement was slightly worse for men (Kappa: 0.43) than for women (Kappa: 0.56). The stratified analysis by age category and education level did not show any specific trend (Additional files 14 and 15). Using self-reported data, the prevalence of hypertension was significantly underestimated and only 45% of the BHIS participants with a measured hypertension were detected (Table 3). The high specificity rates indicate however that self-reported hypertension is a reliable indicator to rule out the existence of hypertension.

For hypercholesterolemia, there was a very poor agreement between self-reported and measured data, with a Kappa coefficient of 5% (Fig. 2). The agreement was slightly worse for men, older people and high educated people (Additional files 16 and 17). Using self-reported data allowed to detect only 22% of the BHIS participants with a measured hypercholesterolemia. By contrast, the high specificity rates indicate that self-reported hypercholesterolemia is a reliable indicator to rule out the existence of hypercholesterolemia.

### Correction for measurement error

#### Regression calibration

With regression models based on the self-reported health condition, age, sex and education level, the measured height and weight could be predicted with relatively good accuracy (R^2^: 93% for height, R^2^: 95% for weight). The accuracy of the model for hypertension was relatively moderate (AUC: 86%) and poor for the hypercholesterolemia model (AUC: 65%). Using predicted values instead of self-reported data yielded higher estimates of people suffering from overweight (+ 8% relative increase), obesity (+ 12%), hypertension (+ 24%) and hypercholesterolemia (+ 36%). Forest plots of the estimates of the regression models for height, weight, hypertension and hypercholesterolemia are available in Additional file 18.

#### Multiple imputation for measurement error (mime)

The missing data pattern of the variables (age, sex, education level and self-reported risk factors) of the merged BELHES/BHIS 2018 dataset is visualized in Additional file 1.

**Fig. 3:**
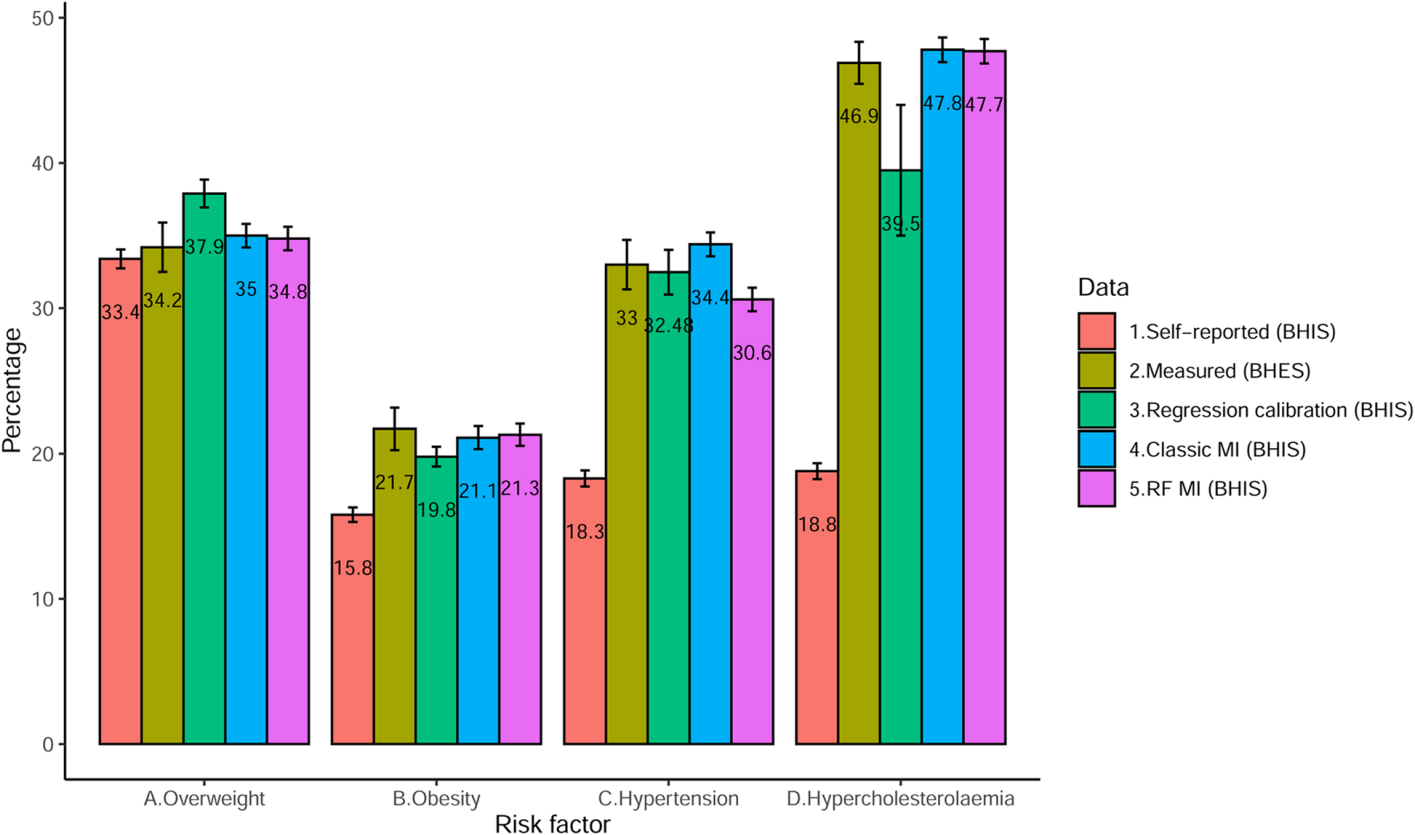
Prevalence estimates of overweight, obesity, hypertension and hypercholesterolemia in Belgium using self-reported, measured and adjusted 2018 BHIS data. Classical MI: classical multiple imputation. RF MI: random-forest multiple imputation. Regression calibration and multiple imputation model included age, sex, education level and the self-reported health conditions. Error bars represent one standard deviation of uncertainty of the estimates

In Fig. 3, the prevalence estimates of overweight, obesity, hypertension and hypercholesterolemia were compared in the different datasets BHES 2018 and BHIS 2018 adjusted with the three correction methods. The convergence of the classic and random-forest multiple imputation is visualized in Additional file 19.

Prevalence estimates based on the predicted values (regression calibration) and the multiply imputed clinical values (random-forest and classic multiple imputation) were closer to their BELHES clinical counterparts than were the BHIS estimates based on self-reported data. Furthermore, for overweight, obesity and hypertension, prevalence estimates based on the adjusted datasets (using regression calibration and multiple imputation) had smaller estimated standard errors than those based solely on the BELHES clinical data (Table 4). By contrast, for hypercholesterolemia, which had a poor model accuracy, regression calibration was less effective than multiple imputation (with standard errors larger than the one obtained in the BELHES measured data).

**Table 4:** Ratio of estimated standard errors: BELHES 2018 clinical data/adjusted BHIS 2018 data

	Regression calibration	Classic multiple imputation	Random-forest multiple imputation
Overweight	1.77	2.10	2.10
Obesity	2.13	1.86	1.93
Hypertension	1.10	2.07	2.10
Hypercholesterolemia	0.32	1.71	1.73

*BHIS* Belgian health interview survey, *BELHES* Belgian Health examination survey

**Fig. 4:**
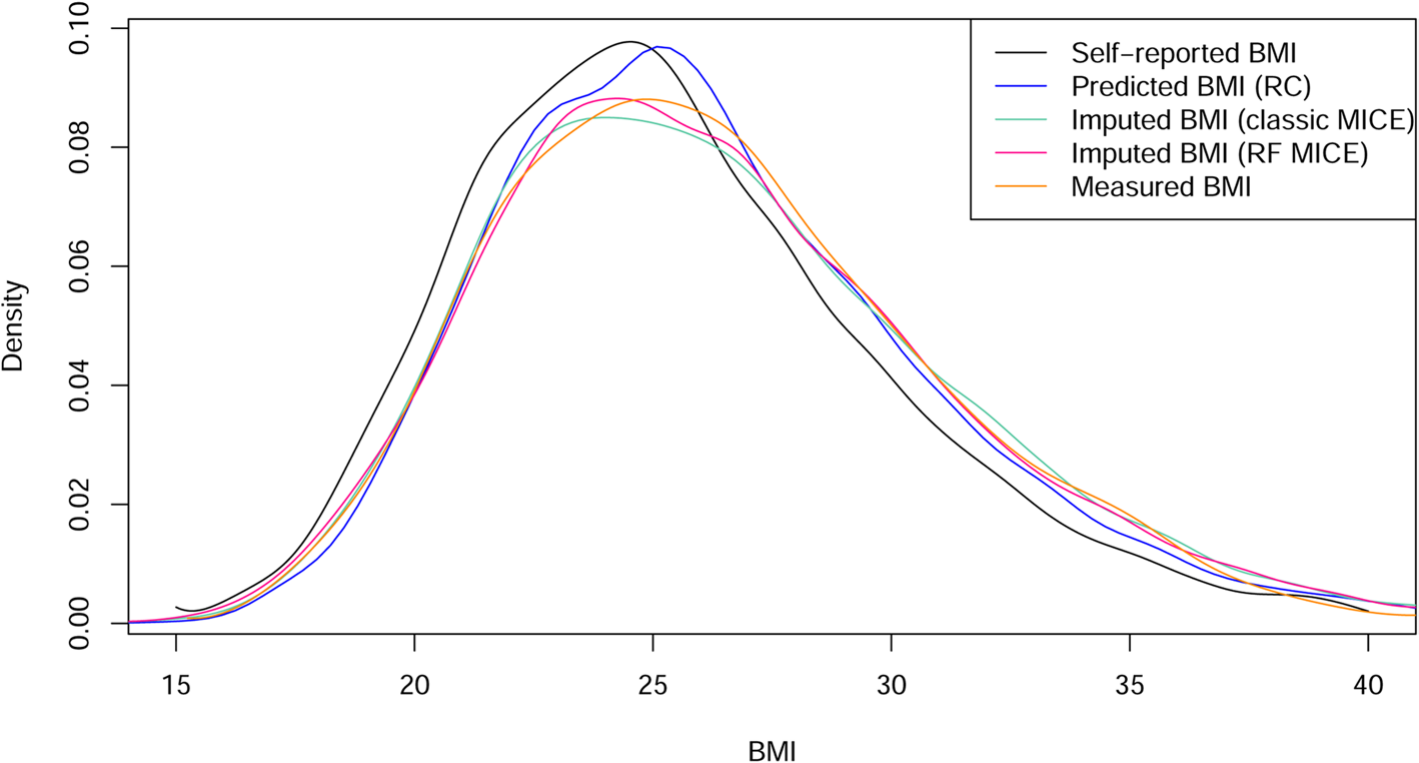
BMI distribution using self-reported, measured and adjusted 2018 BHIS data. For the imputed BMI, only the first imputed dataset was represented for more visibility

By looking at the distribution of BMI in the different adjusted datasets, it appears that the distribution of the imputed BMI (random-forest and classic imputation) is the best approximate of the distribution of the measured BMI (Fig. 4).

#### Sensitivity analyses

Sensitivity analyses included a wider set of variables in the imputation model such as income, smoking status, handicap or chronic disease (variables listed in Additional file 2). Prevalence estimates and standard errors obtained from the multiply imputed datasets using the wider set of variable were similar to the one obtained using the small set of variables. In addition, the random-forest multiple imputation model using the small set of variables was applied to the merged BHIS data from 2008, 2013 and 2018 (*n* = 27,536). The imputation model provided valid prevalence rates for the previous BHIS waves 2008 and 2013, assuming that the trend in the prevalence estimates remained approximately the same across the last ten years (Additional file 20). It is also interesting to note that the imputation-based analysis provided valid results for hypercholesterolemia including for the year 2008, for which the self-reported data on hypercholesterolemia was not available. Finally, applying the imputation model to the larger dataset including the three BHIS waves resulted in even smaller standard errors (Additional file 21).

## Discussion

### Main findings

Consistent with previous literature, this study showed an underestimation of the prevalence of obesity, hypertension and hypercholesterolemia based on self-reported data [3, 5–7, 10, 13, 14, 16, 46–50]. The observed under-reporting for weight and over-reporting for height, resulting in a underestimation of the BMI, is a general trend observed in many studies although the degree of the trend varies for men and women and the characteristics of the population being examined [6].

The self-reported prevalence of obesity was six percentage points lower for both men and women but did not show an underestimation of the prevalence of overweight, confirming a higher misreporting bias among people with obesity. In a literature review, Maukonen et al. reported similar results with an underestimation of obesity ranging from 0.7% points to 13.4% points [3] and highly heterogeneous results regarding overweight prevalence [3, 51]. The higher self-reporting bias observed in specific subgroups such as women, older people and participants with obesity has been observed in several previous studies [3, 52–56]. Social desirability, leading people to report values that are closed to their ideal, could partially explain this observation [57]. The over-reporting of height in older people may be due to the fact that people have their height measured over a decade ago but became shorter with age.

The weak validity of self-reported hypertension observed in our study is in line with several study results showing that approximately half of patients with hypertension would not be identified by self-reporting in epidemiological studies [7–10, 13, 16]. In the same trend, our study shows that the prevalence of elevated total serum cholesterol based on measurements was much higher (47%) than the self-reported prevalence of hypercholesterolemia (21%). Using self-reported data only, 78% of the population suffering from hypercholesterolemia was missed compared with data from objective measurements. Similar results were obtained in previous studies [13, 42]. Specificity, by contrast, provided accurate results for all risk factors (> 99% for obesity and hypertension and 83% for hypercholesterolemia), which means that self-reported measures can be considered reliable to rule out the existence of the risk factors in the population.

The inaccuracy of self-reported hypertension and hypercholesterolemia could be explained by the fact that they are usually asymptomatic and remain therefore easily undetected. This might also be related to clinicians using different threshold values to identify and communicate the risk factor. For hypertension for example, it is worth noting that some medical staff may continue to use the old diagnosis criterion (which changed in 1999 from 160/95 to 140/90 mmHg) and thereby wrongly classify patients with hypertension as not having the disease. Regarding hypercholesterolemia, people may only report it if their physician told them they had a high cholesterol risk factor, which is in fact the ratio total cholesterol/HDL cholesterol. Even if an elevated total cholesterol (defined as total cholesterol > 190 mmol/l) is a WHO recommended indicator to monitor NCDs [58], it does not represent in itself a risk factor.

The frequency of physician visits, educational level, urban living and access to healthcare have been identified as factors associated with the accuracy of self-reporting hypertension and hypercholesterolemia [7]. The underestimation of self-reported hypertension and hypercholesterolemia demonstrates that screening for those cardiovascular risk factors needs to be strengthened and population awareness of early detection of those risk factors to be increased.

With both regression calibration and multiple imputation, adjusted estimation of height, weight, hypertension and hypercholesterolemia in the BHIS 2018 allowed to generate national prevalence rates that were closer to their BELHES clinical counterparts. For overweight, obesity and hypertension, all methods provided smaller standard errors than those obtained with clinical data alone. However, for hypercholesterolemia, for which the regression model’s accuracy was poor, MIME has better performance than regression calibration. This result should however be taken with caution because the random-forest MIME might potentially give underestimated standard errors as a result of ignoring the uncertainty in the imputation models fitted via random-forest [34]. One theoretical reason for expecting MIME to perform better than regression calibration is because MIME uses the measured risk factor when it is available, rather than imputing it, whereas regression calibration always predicts the measured risk factor from the self-reported risk factor [23]; another reason is that imputations obtained by MIME resemble real data better by acknowledging that real data vary around the (predicted) mean.

Sensitivity analyses demonstrated that the imputation model based on a small set of variables (self-reported health condition, age, sex and education) was largely sufficient to correct the measurement error in the BHIS data. Since the self-reported measure is such a strong predictor, additional variables in the imputation model had little influence and did not improve the efficiency of the imputations. Furthermore, by applying the imputation model to the complete BHIS dataset from 2008 to 2018, results shows that the method could correctly predict the missing clinical values for the previous BHIS waves 2008 and 2013.

### Regression calibration or multiple imputation?

While regression calibration is quite easy to implement, this method should however be used with caution because of its inherent limitations. First, this is so because predictive equations to correct for self-reporting bias will only work if the percentage of explained variance is very high [25]. Secondly, regression calibration does not take into account the uncertainty in the estimated prediction because the method is based on single predicted values, which may result in potentially biased standard errors [18]. Even if the method may be appropriate to model the population distribution of the risk factor, this method is not recommended if the researcher is interested in using the adjusted risk factor as a predictor variable for modelling disease [27]. Finally, with regard to the bias related to self-reported BMI, studies have shown that predictive equations were unsuitable as correction methods because they had a systematic downward bias [24, 26]. Because the relationship of self-reported BMI to measured BMI is characterized by a “flat slope syndrome” (over reporting of low values and underreporting of high values), the self-reported bias in BMI is highly correlated with measured BMI [26].

Among the different methods explored in this study, the random-forest multiple imputation proved to be preferred to correct the self-reported bias in the BHIS. Although this method is only applicable if a validation study is available (where a “true” exposure is measured in a subsample) [59], its offers numerous advantages. Unlike the regression calibration, the random-forest multiple imputation has the advantage to explicitly account for the uncertainty in the predicted clinical measurement and, hence, produce more reliable statistical inferences [25]. Additionally, MIME allows to easily handle the missing data problem of all covariates in the same process, which increases the statistical power when assessing the risk factor disease association in survey data. Finally, the random-forest MIME brings two additional benefits compared to classic multiple imputation. Unlike the standard imputation approach, the random forest-based imputation handles data with complex interactions or non-linearity and does not assume normality or require specification of parametric models. Secondly, because of this additional complexity, the random forest-based imputation does not suffer from the “congeniality” problem that it must obey the form of the final analysis model. This assumption required by the standard imputation approach may not be met if the goal is to allow researchers to use these imputations in subsequent analyses. In view of this, the random-forest MIME was the chosen method to impute 10 clinical values of the risk factors of interest for all BHIS participants from 2008 to 2018. While researchers are aware that measurement error related to self-reported data could affect the results of their studies, very few adjusted their analysis for the error. Furthermore, they often do not provide a complete discussion of the potential effects of measurement error on their results*.* By providing 10 imputed clinical values for height, weight, BMI, hypertension and hypercholesterolemia in the BHIS 2008/2013/2018 we aimed to enable secondary analysts to improve their analysis of self-reported BHIS data by using information included in the BELHES. However, caution is needed when using the imputed clinical values. Those imputed values may be used to model an exposure-disease association or to provide prevalence estimates using the Rubin ‘s rule. They should not be used in combination with risk estimates based on unadjusted self-reported data only. For example, to calculate a population attributable fraction (PAF), the risk estimate should not be taken from the literature but rather computed from the adjusted BHIS data. The PAF is used to estimate the burden of a risk factor and is based on the risk estimate of the risk factor and the prevalence of the disease in the population. Calculating a PAF using a risk estimate based on self-reported data and a prevalence of the risk factor based on corrected data would be the same as comparing apples and oranges.

### Strengths and limitations

The main added value of this study resides in the novelty of the approach. To our knowledge, this study is the first to consider a random forest-based multiple imputation to correct the measurement error related to self-reported data in health interview surveys. This has been made possible thanks to the validation sample, the BELHES 2018, where data on self-reported medical conditions could be compared with objective measurements for the same individuals. Furthermore, this study is based on a nationwide, large scale population survey, using standardized methods, regarding the sampling, questionnaires and measurement protocols, which makes our results comparable across countries.

The findings of this study must nevertheless be seen in the light of some limitations. The definitions and selected cut-off values for the measured risk factors could be questioned, since according to the reference standards considered, results on the agreement with self-reported data may vary substantially. If WHO categories are widely used to determine obesity, a higher heterogeneity of gold standards was found to diagnose hypertension and hypercholesterolemia across studies. In our study, hypertension was defined as a systolic blood pressure ≥ 140 mmHg or a diastolic blood pressure > 90 mmHg or medication use for hypertension; and hypercholesterolemia as a total cholesterol level > 190 mg/dl (> 5 mmol/l). In other studies, hypertension was sometimes diagnosed using a 160/90 cut-off and reference ranges for hypercholesterolemia varied from 5 to 6.5 mmol/l. Medication was furthermore not always taken into account in the definition of the risk factor [13]. In our definition of the measured hypercholesterolemia (Table 1), we decide to not include the use of medication because statins are often used as preventive treatment. Regardless of the selected cut-off, the prevalence of specific risk factors in health examination surveys could still be over- or under-estimated because measurements are taken on a single occasion while a medical diagnosis of hypertension or hypercholesterolemia is generally based on several subsequent measurements. In self-reported data, the format of questions may also impact the validity results. In our study, the self-reported health condition relied on the question: “Did you suffer from…in the last 12 months?”, but in some other studies, only diagnosed conditions or conditions that a health professional had ‘told’ about were enquired. The main challenge of a diagnostic method is to obtain a satisfactory balance between high sensitivity and high specificity, yielding a minimum of both false positives and false negatives. Sensitivity estimates related to self-reported obesity, hypertension and hypercholesterolemia are particularly important in health interview surveys, as they ensure identification of the largest number of people at risk of developing NCDs. A second limitation of our study is related to the underrepresentation of low educated people in the validation sample. Because of the second stage recruitment of the BELHES, this underrepresentation, which was already present in the BHIS, was reinforced. Unfortunately, educational level was not taken into account in the survey weights, because this information was not available. Thirdly, the imputed clinical values in the BHIS 2008/2013/2018 were all based on the available validation sample BELHES 2018, which implies that our analysis assumes self-reporting bias not to change over time. This assumption may however not be met, since the awareness of one’s own condition may have increased due to the common use of digital devices at home for measuring blood pressure and the wider availability of blood glucose measurements in pharmacies. Subsequent BELHES data in the coming years, when available, should therefore be used to update the imputed clinical values in the following BHIS datasets.

Finally, it is important to have in mind that, in epidemiology, measurement error in confounders might be even more challenging than measurement error in exposure. Measurement error in confounders can lead to overestimation of exposure–disease associations whereas measurement error in exposures typically dilutes the associations. Future analysis could therefore be conducted to extend the MIME correction to other important self-reported risk factors or confounders such as smoking or diabetes in the BHIS data.

## Conclusions

Obesity, hypertension and hypercholesterolemia are leading biomedical risk factors of NCDs with surveillance often based on self-reported data. With a general increase in these risk factors rates in Belgium it is of paramount importance to obtain accurate prevalence data to correctly assess the effectiveness of NCD prevention programs. Results of this study confirm that using self-reported data alone leads to a severe underestimation of the prevalence of obesity, hypertension and hypercholesterolemia in Belgium. By exploring different approaches to correct for measurement error, this study shows how information from the BHIS and BELHES 2018 can be combined to provide a valid correction of those risk factors. Both regression calibration and MIME techniques generate accurate national prevalence rates of these risk factors, that could in turn be used by decision makers to allocate resources and set priorities in health. Our results suggest however that the random-forest multiple imputation is the most appropriate choice to correct the measurement error related to self-reported data in health interview surveys. Besides its ability to handle data with complex interaction or non-linearity, the technique has the advantage that it does not require to specify an imputation model which is particularly useful to allow secondary analysts to improve their analysis of self-reported data by using information included in the BELHES. Whenever feasible, combined information from health interview survey and measurements should be used in risk factor monitoring.

## Supplementary Information

**Additional file 1.** Missing data pattern of the merged Belgian health interview survey/Belgian health examination survey 2018 dataset.

**Additional file 2.** List of variables from the wider set of variables included in the imputation model.

**Additional file 3.** Description of the population.

**Additional file 4.** Bland-Altman plot for analysis of agreement between self-reported and measured height (by gender).

**Additional file 5.** Bland-Altman plot for analysis of agreement between self-reported and measured height (by age category).

**Additional file 6.** Bland-Altman plot for analysis of agreement between self-reported and measured height (by education level).

**Additional file 7.** Bland-Altman plot for analysis of agreement between self-reported and measured weight (by gender).

**Additional file 8.** Bland-Altman plot for analysis of agreement between self-reported and measured weight (by age category). 

**Additional file 9.** Bland-Altman plot for analysis of agreement between self-reported and measured weight (by education level).

**Additional file 10.** Bland-Altman plot for analysis of agreement between self-reported and measured BMI (by age category).

**Additional file 11.** Bland-Altman plot for analysis of agreement between self-reported and measured BMI (by education level).

**Additional file 12.** Prevalence of overweight, obesity, hypertension and hypercholesterolemia using self-reported and measured data (by age).

**Additional file 13.** Prevalence of overweight, obesity, hypertension and hypercholesterolemia using self-reported and measured data (by education level).

**Additional file 14.** Confusion matrix comparing self-reported and measured high blood pressure (by age category).

**Additional file 15.** Confusion matrix comparing self-reported and measured high blood pressure (by education level).

**Additional file 16.** Confusion matrix comparing self-reported and measured hypercholesterolemia (by age category).

**Additional file 17.** Confusion matrix comparing self-reported and measured hypercholesterolemia (by education level).

**Additional file 18.** Estimates of the regression models for height, weight, hypertension and hypercholesterolemia. 

**Additional file 19.** Mean and standard deviation of the synthetic values plotted against iteration number for the classic and Random-forest multiply imputed 2018 BHIS data.  

**Additional file 20.** Prevalence estimates of overweight, obesity, hypertension and hypercholesterolemia in Belgium using self-reported, measured and adjusted BHIS data for  2008, 2013, and 2018. 

**Additional file 21.** Ratio of estimated standard errors: BELHES 2018 clinical/adjusted BHIS 2008-2013-2018.

## Data Availability

The data that support the findings of this study are not publicly available. Data are however available from the authors upon reasonable request and with specific permission (https://www.sciensano.be/en/node/55737/health-interview-survey-microdata-request-procedure). Legal restrictions make that BHIS and BHES data can only be communicated to other parties if an authorization is obtained from the sectoral committee social security and health of the Belgian data protection authority.
