# Supplementary material for: Using random-forest multiple imputation to address bias of self-reported anthropometric measures, hypertension and hypercholesterolemia in the Belgian health interview survey
Source: BMC Med Res Methodol. 2023 Mar 25;23:69. doi: 10.1186/s12874-023-01892-x (PMC10040120; doi:10.1186/s12874-023-01892-x)
Supplement: Supplementary file 1 — Additional file 1. Missing data pattern of the merged Belgian health interview survey/Belgian health examination survey 2018 dataset. [file 12874_2023_1892_MOESM1_ESM.pdf]

Additional file 1. Missing data pattern of the merged Belgian health interview survey/Belgian health examination survey 2018 dataset

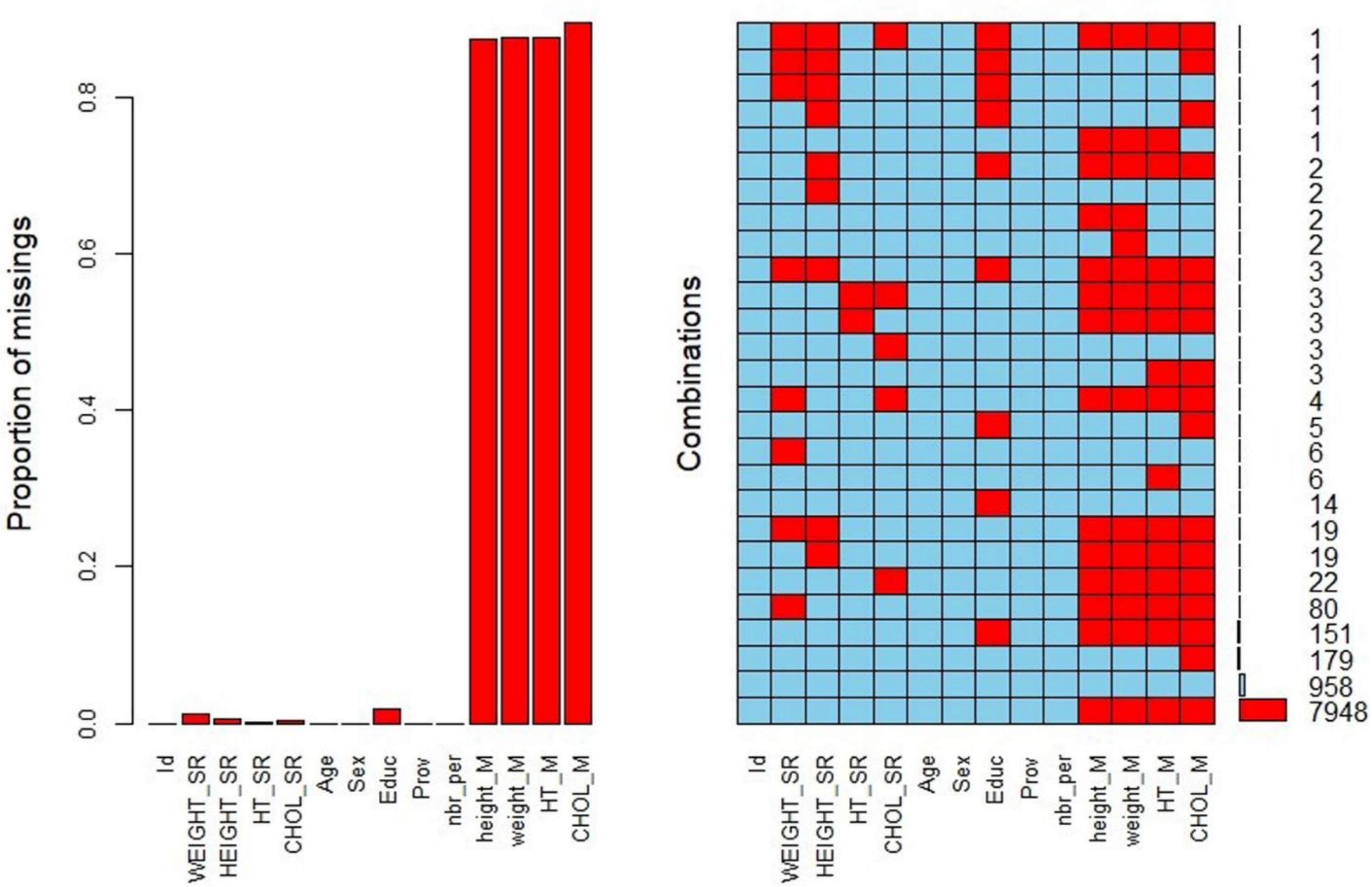


Weight_SR: SR weight, Height_SR: SR height, HT_SR: SR hypertension, CHOL_SR: SR hypercholesterolemia, Educ: Education level, nbr_pers: Number of persons in the household, height_M: measured height, weight_M: measured weight, HT_M: measured hypertension, CHOL_M: measured hypercholesterolemia.
