# Supplementary material for: Using random-forest multiple imputation to address bias of self-reported anthropometric measures, hypertension and hypercholesterolemia in the Belgian health interview survey
Source: BMC Med Res Methodol. 2023 Mar 25;23:69. doi: 10.1186/s12874-023-01892-x (PMC10040120; doi:10.1186/s12874-023-01892-x)
Supplement: Supplementary file 2 — Additional file 2. List of variables from the wider set of variables included in the imputation model. [file 12874_2023_1892_MOESM2_ESM.pdf]

Additional file 2. List of variables from the wider set of variables included in the imputation model

Socio-economic factors	Age, gender, highest household educational level**,** household composition, country of birth, civil status, household income
Lifestyle factors	Smoking status, past 12 months frequency of alcohol consumption, leisure time physical activity, frequency of eating fruit, frequency of eating vegetables, frequency of drinking soft drinks, frequency of eating snack, drinking the daily recommended amount of water (1 liter)
Health condition	Diabetes, cancer, multimorbidity, number of chronic condition, chronic condition, handicap, heart disease
SR risk factors of interest	Hypertension, hypercholesterolemia, BMI, height, weight

SR: self-reported
