# Supplementary material for: Using random-forest multiple imputation to address bias of self-reported anthropometric measures, hypertension and hypercholesterolemia in the Belgian health interview survey
Source: BMC Med Res Methodol. 2023 Mar 25;23:69. doi: 10.1186/s12874-023-01892-x (PMC10040120; doi:10.1186/s12874-023-01892-x)
Supplement: Supplementary file 3 — Additional file 3. Description of the population. [file 12874_2023_1892_MOESM3_ESM.pdf]

Additional file 3. Description of the population

	Prevalence (95% CI)	N	Mean (95% CI)	Median
**Socio-economic status**				
Age				
18/24	9.98 [9.04;11]	745		
25/44	32.4 [30.9;34]	2916		
45/64	34.4 [33;36]	3395		
>65	23.2 [21.9;25]	2375		
NA's		8		
Sex				
Men	49 [48;50]	4916		
Women	51.3 [50;52]	4523		
Household educational level				
Higher education	47.6 [45.6;49.6]	4503		
Higher secondary	32.7 [30.9;35]	2798		
Lower secondary	13.2 [12-14]	1246		
No diploma/primary	6.51 [5.76;7]	713		
NA's		179		
Household composition				
Couple with child(ren)	37.8 [35.9;4]	3377		
Couple without child(ren)	28.4 [26.8;3]	2598		
1 parent with child(ren)	9.61 [8.55;11]	837		
Single	19.6 [18.4;21]	2151		
Country of birth				
Belgian	82.7 [81.5;84]	7125		
Non belgian EU	6.91 [6.24;8]	1057		
Non belgian non EU	10.37 [9.41;11]	1250		
NA's		7		
Civil status				
Married/cohabiting	54.5 [52.9;56]	5204		
Divorced	9.71 [8.87;11]	973		
Widow	7.38 [6.7;8]	728		
Single	28.4 [27;30]	2534		
NA's		0		
Reported household income (quintile)				

1	12 [11;13]	1101		
2	14.9 [13.6;16]	1256		
3	19.5 [17.9;21]	1545		
4	25.1 [23.4;27]	1934		
5	28.5 [26.5;31]	2076		
NA's		1527		
**Lifestyle**				
Smoking status				
Never smoker	56.2 [54.6;58]	4238		
Former smoker	23.9 [22.6;25]	1860		
Occasional smoker	40.4 [34.6;50]	322		
Daily smoker	15.8 [14.6;17]	1166		
NA's		1823		
Past 12 months frequency of alcohol consumption			5.03 [4.95;5.11]	4
NA's		1788		
Leisure time physical activity				
Light	52.1 [50.5;54]	3791		
Sedentary	29.5 [28;31]	2231		
Intensive	18.4 [17.1;2]	1234		
NA's		2183		
Frequency of eating vegetables				
Once or more a day	76.2 [74.8;78]	7007		
Never	0.66 [0.45;1]	61		
Less than once a week	1.58 [1.28;2]	163		
1 to 3 times a week	8.19 [7.40;9]	853		
4 to 6 times a week	13.4 [12.4;14]	1349		
NA's		5		
Frequency of eating fruit				
Once or more a day	55.3 [53.7;57]	5346		
Never	3.85 [3.31;4]	327		
Less than once a week	8.9 [8.06;1]	773		
1 to 3 times a week	18.7 [17.6;2]	1721		
4 to 6 times a week	13.2 [12.2;14]	1265		
NA's		7		

Drinking the daily recommended amount of water (1 litre)				
yes	37.2 [35.7;39]	3715		
NA's		25		
Frequency of eating snack				
Once or more a day	35.5 [34;37]	3131		
Never	8.51 [7.72;9]	871		
Less than once a week	14.7 [13.7;16]	1469		
1 to 3 times a week	28.4 [27.1;3]	2780		
4 to 6 times a week	12.9 [11.9;14]	1180		
NA's		8		
Frequency of drinking soft drinks				
Once or more a day	20.4 [19.1;22]	1743		
Never	39.4 [37.9;41]	3850		
Less than once a week	18.1 [17;19]	1807		
1 to 3 times a week	16.1 [15;17]	1508		
4 to 6 times a week	5.98 [5.30;7]	525		
NA's		6		
**Health status (self-reported status)**				
SR BMI		9300	25.67 [25.53;25.81]	25.07
NA's		139		
SR BMI (Category)				
0-18.5	2.93 [2.45;3]	270		
18.5-25	47.8 [46.4;49]	4334		
25-30	33.4 [32.1;35]	3197		
30,Inf	15.8 [14.8;17]	1499		
NA's		139		
SR Cholesterol	18.8 [17.7;2]	1779		
NA's		33		
SR Hypertension	18.3 [17.2;19]	1787		
NA's		6		
SR Diabete	6.08 [5.48;7]	624		
NA's		3		
SR Heart disease	4.77 [4.20;5]	447		
NA's		2		

SR Cancer	2.47 [2.07;3]	223		
NA's		1		
Multimorbidity	15.8 [14.8;17]	1534		
NA's		20		
SR Chronic condition	29.9 [28.5;31]	2382		
NA's		1712		
Number of SR chronic conditions				
0	59.4 [57.9;61]	5523		
1	24.8 [23.6;26]	2362		
2	11.0 [10.1;12]	1067		
3	4.8 [4.26;5.0]	467		
NA's		20		
SR Handicap				
Limited	23.2 [21.9;25]	1883		
Not limited	76.8 [75.4;78]	5824		
NA's		20		
**Health status (Measured variables)**				
Measured BMI		1179	26.53 [26.14;26.92]	25.75
NA's		8260		
Measured waist circumference (cm)		1014	92.31 [91.15;93.47]	92
NA's		8425		
Measured glucose (fasting plasma glucose)		974	92.44 [90.87;94]	89.3
NA's		8465		
Measured Diabetes (Raised blood glucose/diabetes based on fasting glucoseHbA1c or use of diabetes medication)	9.7 [7.58;12]	105		
NA's		8377		
Measured hypertension (Systolic blood pressure ≥140 mmHg or diastolic	31.6 [28.2;35]	382		

blood pressure >90 mmHg or medication use for hypertension				
NA's		8265		
Measured systolic blood pressure (mmHg)		1179	120.13 [118.72;121.53]	118.5
NA's		8260		
Measured dyastolic blood pressure (mmHg)		1179	77.51 [76.63;78.39]	77
NA's		8260		
Measured cholesterolemia (Total serum cholesterol > 190 mg/dl)	48.7 [44.6;53]	490		
NA's		8444		
Measured total cholesterol (mg/dL)		490	192.24 [188.95;195.53]	189.3
NA's		8444		
**Environment**				
Green surrounding % (1000m)		9439	39.56 [39.46;39.66]	31.84
PM_{10} exposure (µg/m3)		9439	19.81 [19.71;19.91]	20.27
$PM_{2.5}$ exposure (µg/m^3)		9439	12.16 [12.1;12.22]	12.81
BC exposure (µg/m^3)		9439	0.95 [0.94;0.96]	0.93
$N0_2$ exposure (µg/m3)		9439	18.51 [18.33;18.69]	18.45
