# Supplementary material for: Using random-forest multiple imputation to address bias of self-reported anthropometric measures, hypertension and hypercholesterolemia in the Belgian health interview survey
Source: BMC Med Res Methodol. 2023 Mar 25;23:69. doi: 10.1186/s12874-023-01892-x (PMC10040120; doi:10.1186/s12874-023-01892-x)
Supplement: Supplementary file 8 — Additional file 8. Bland-Altman plot for analysis of agreement between self-reported and measured weight (by age category). [file 12874_2023_1892_MOESM8_ESM.pdf]

Additional file 8. Bland-Altman plot for analysis of agreement between self-reported and measured weight (by age category)

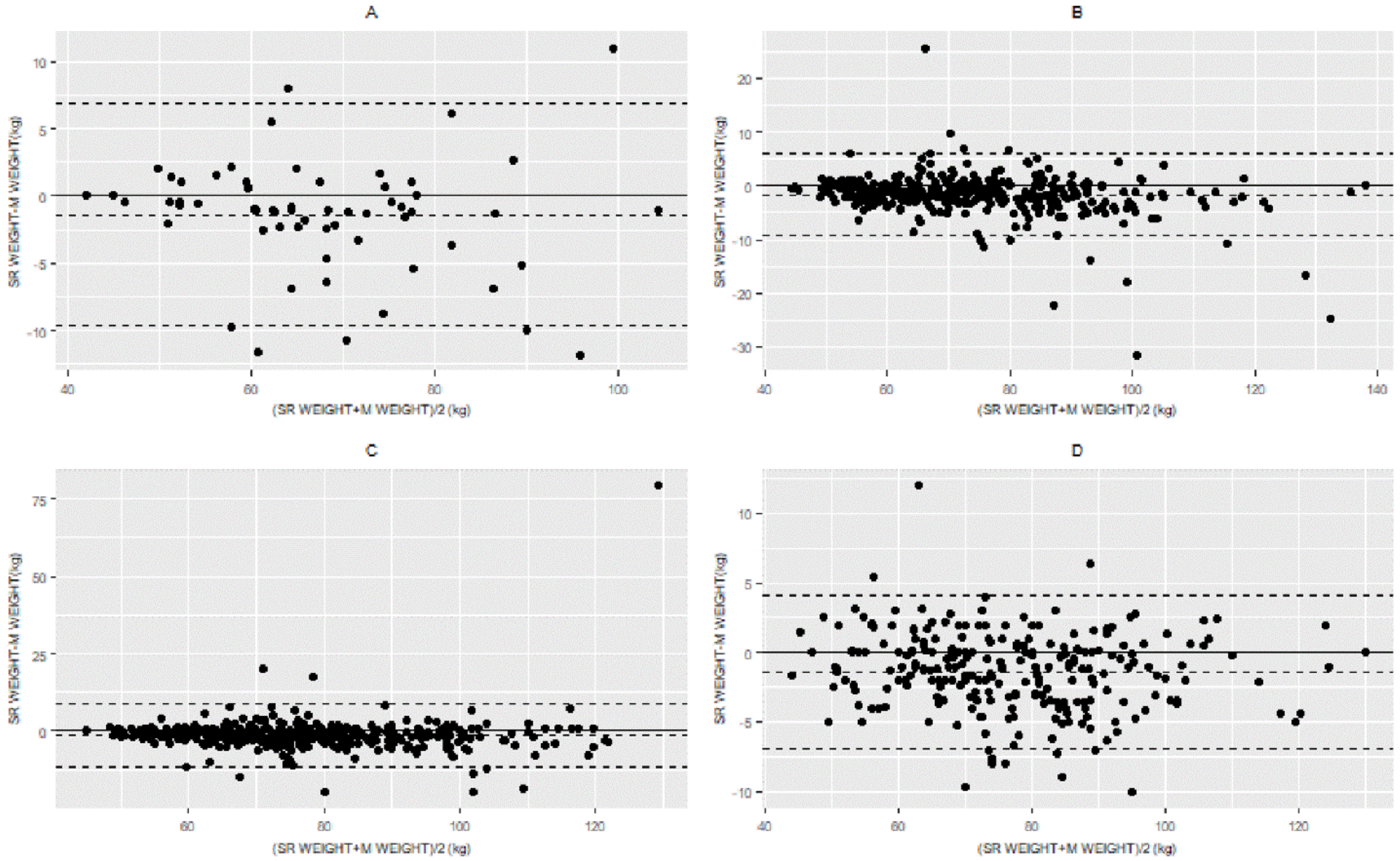

A: 18 to 24, B: 25/44, C: 45/64, D:>65. The solid line represents the mean difference. The dashed lines represent upper and lower of agreement (mean difference +/- 2 standard deviations).
