# Supplementary material for: Using random-forest multiple imputation to address bias of self-reported anthropometric measures, hypertension and hypercholesterolemia in the Belgian health interview survey
Source: BMC Med Res Methodol. 2023 Mar 25;23:69. doi: 10.1186/s12874-023-01892-x (PMC10040120; doi:10.1186/s12874-023-01892-x)
Supplement: Supplementary file 12 — Additional file 12. Prevalence of overweight, obesity, hypertension and hypercholesterolemia using self-reported and measured data (by age). [file 12874_2023_1892_MOESM12_ESM.pdf]

Additional file 12. Prevalence of overweight, obesity, hypertension and hypercholesterolemia using self-reported and measured data (by age)

	Overweight (%)				Obesity (%)			
	18-24	26-44	45-64	>65	18-24	26-44	45-64	>65
M.prevalence %	22	30	38	45	6	16	23	30
	11;34	24;35	32;42	38;51	1;18	11;22	18;28	23;36
SR. prevalence	17	29	39	42	5	13	16	20
95%IC	8;30	24;34	34;44	36;49	1;17	8;18	11;21	13;27
Sensitivity (%)	71	79	80	72	75	73	66	64
Specificity (%)	98	92	85	82	99	99	98	99
VPP	91	82	76	77	99	92	93	98
VPN	92	91	87	79	98	95	90	87
	Hypertension (%)				Hypercholesterolemia (%)			
	18-24	26-44	45-64	>65	18-24	26-44	45-64	>65
M.prevalence %	6	12	36	65	37	38	60	48
95% IC	1;11	9;15	32;41	69;71	25;49	33;43	56;66	41;56
SR.prevalence%	1	6	18	32	12	6	24	36
95%IC	0;4	4;9	15;22	26;38	5;20	5;9	20;28	60;44
Sensitivity (%)	0	39	44	49	18	14	24	30
Specificity (%)	98	98	96	99	92	97	77	58
VPP	0	73	88	99	57	65	61	41
VPN	93	92	75	51	65	75	40	47
