# Supplementary material for: Using random-forest multiple imputation to address bias of self-reported anthropometric measures, hypertension and hypercholesterolemia in the Belgian health interview survey
Source: BMC Med Res Methodol. 2023 Mar 25;23:69. doi: 10.1186/s12874-023-01892-x (PMC10040120; doi:10.1186/s12874-023-01892-x)
Supplement: Supplementary file 13 — Additional file 13. Prevalence of overweight, obesity, hypertension and hypercholesterolemia using self-reported and measured data (by education level). [file 12874_2023_1892_MOESM13_ESM.pdf]

Additional file 13. Prevalence of overweight, obesity, hypertension and hypercholesterolemia using self-reported and measured data (by education level)

	Overweight (%)				Obesity (%)			
	High	High s.	Low s.	No ed	High	High s.	Low s.	No ed
M.prevalence %	34	35	38	34	17	31	23	17
	29;38	26;46	33;43	30;38	13;21	22;42	17;29	13;21
SR. prevalence	33	39	37	33	12	22	17	12
95%IC	29;37	31;50	32;43	29;39	8;16	13;33	12;23	8;16
Sensitivity (%)	77	87	77	72	66	73	70	64
Specificity (%)	90	86	88	82	99	99	98	99
VPP	81	77	80	77	93	99	92	98
VPN	88	92	86	79	93	89	91	87
	Hypertension (%)				Hypercholesterolemia (%)			
	High	High s.	Low s.	No ed	High	High s.	Low s.	No ed
M.prevalence %	50	50	35	25	56	47	49	49
95% IC	37;53	18;33	30;41	22;14	41;70	37;58	43;55	41;53
SR.prevalence%	25	25	19	12	34	26	20	17
95%IC	16;37	18;33	15;23	9;14	21;50	18;35	15;24	14;21
Sensitivity (%)	46	45	50	41	26	26	19	19
Specificity (%)	96	96	97	98	55	56	79	79
VPP	92	89	91	87	42	43	47	47
VPN	64	63	77	83	37	37	50	50
