# Supplementary material for: Using random-forest multiple imputation to address bias of self-reported anthropometric measures, hypertension and hypercholesterolemia in the Belgian health interview survey
Source: BMC Med Res Methodol. 2023 Mar 25;23:69. doi: 10.1186/s12874-023-01892-x (PMC10040120; doi:10.1186/s12874-023-01892-x)

Additional file 14. Confusion matrix comparing self-reported and measured high blood pressure (by age category)

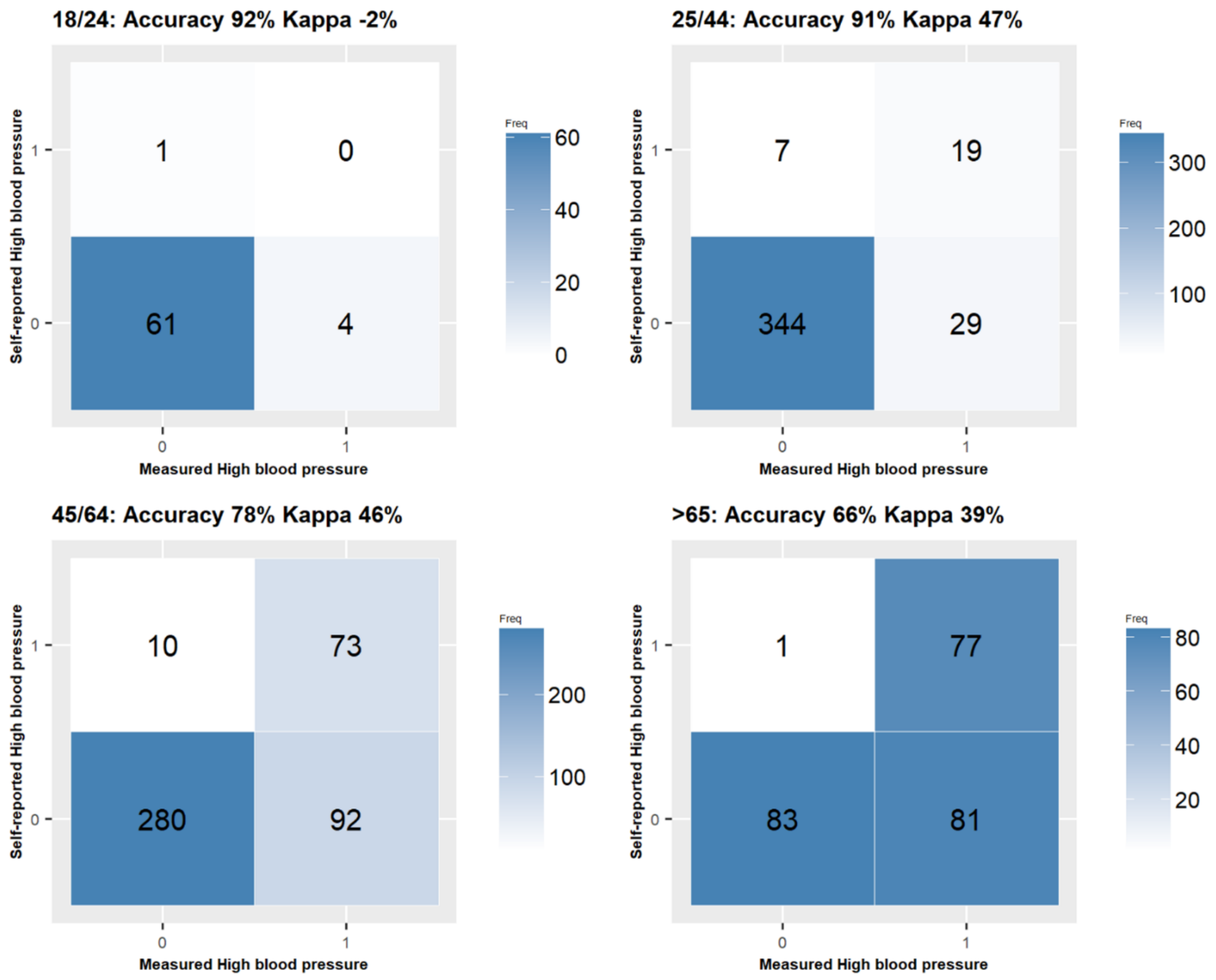

Supplement: Supplementary file 14 — Additional file 14. Confusion matrix comparing self-reported and measured high blood pressure (by age category). [file 12874_2023_1892_MOESM14_ESM.pdf]
