# Supplementary material for: Using random-forest multiple imputation to address bias of self-reported anthropometric measures, hypertension and hypercholesterolemia in the Belgian health interview survey
Source: BMC Med Res Methodol. 2023 Mar 25;23:69. doi: 10.1186/s12874-023-01892-x (PMC10040120; doi:10.1186/s12874-023-01892-x)
Supplement: Supplementary file 18 — Additional file 18. Estimates of the regression models for height, weight, hypertension and hypercholesterolemia. [file 12874_2023_1892_MOESM18_ESM.pdf]

Additional file 18. Estimates of the regression models for height, weight, hypertension and hypercholesterolemia

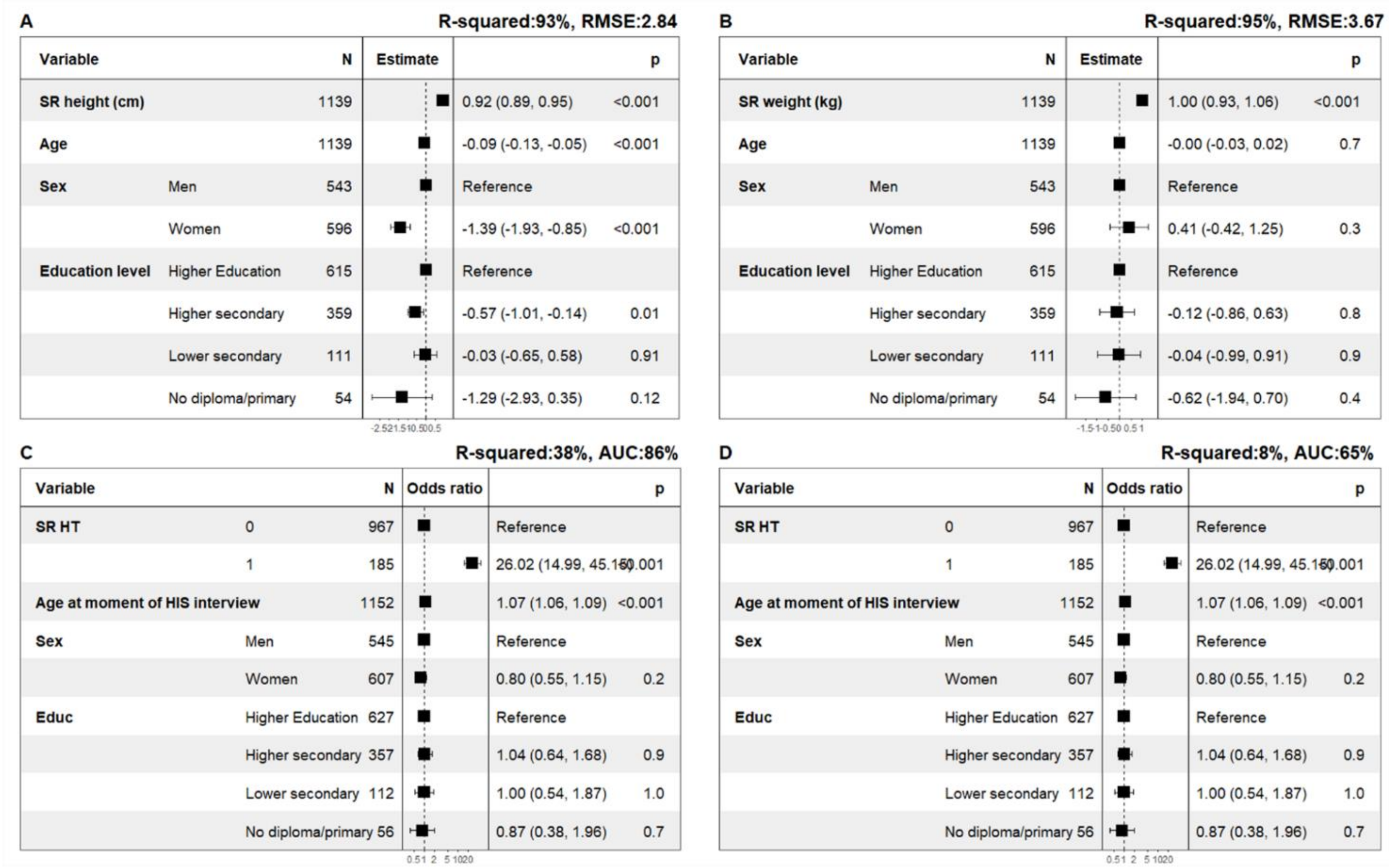

A: Regression model for height, B: regression model for weight, C: regression model for hypertension, D: regression model for hypercholesterolemia. For model A: interaction terms was included between the self-reported height and age category. For model D, interaction terms were included between the self-reported hypercholesterolemia and age category, education level and sex. R^2= coefficient of determination, RMSE: root-mean-squared error, AUC: area under the curve
