# Supplementary material for: Using random-forest multiple imputation to address bias of self-reported anthropometric measures, hypertension and hypercholesterolemia in the Belgian health interview survey
Source: BMC Med Res Methodol. 2023 Mar 25;23:69. doi: 10.1186/s12874-023-01892-x (PMC10040120; doi:10.1186/s12874-023-01892-x)
Supplement: Supplementary file 19 — Additional file 19. Mean and standard deviation of the synthetic values plotted against iteration number for the classic and Random-forest multiply imputed 2018 BHIS data. [file 12874_2023_1892_MOESM19_ESM.pdf]

Additional file 19. Mean and standard deviation of the synthetic values plotted against iteration number for the classic and Random-forest multiply imputed 2018 BHIS data.

« Classic » MICE

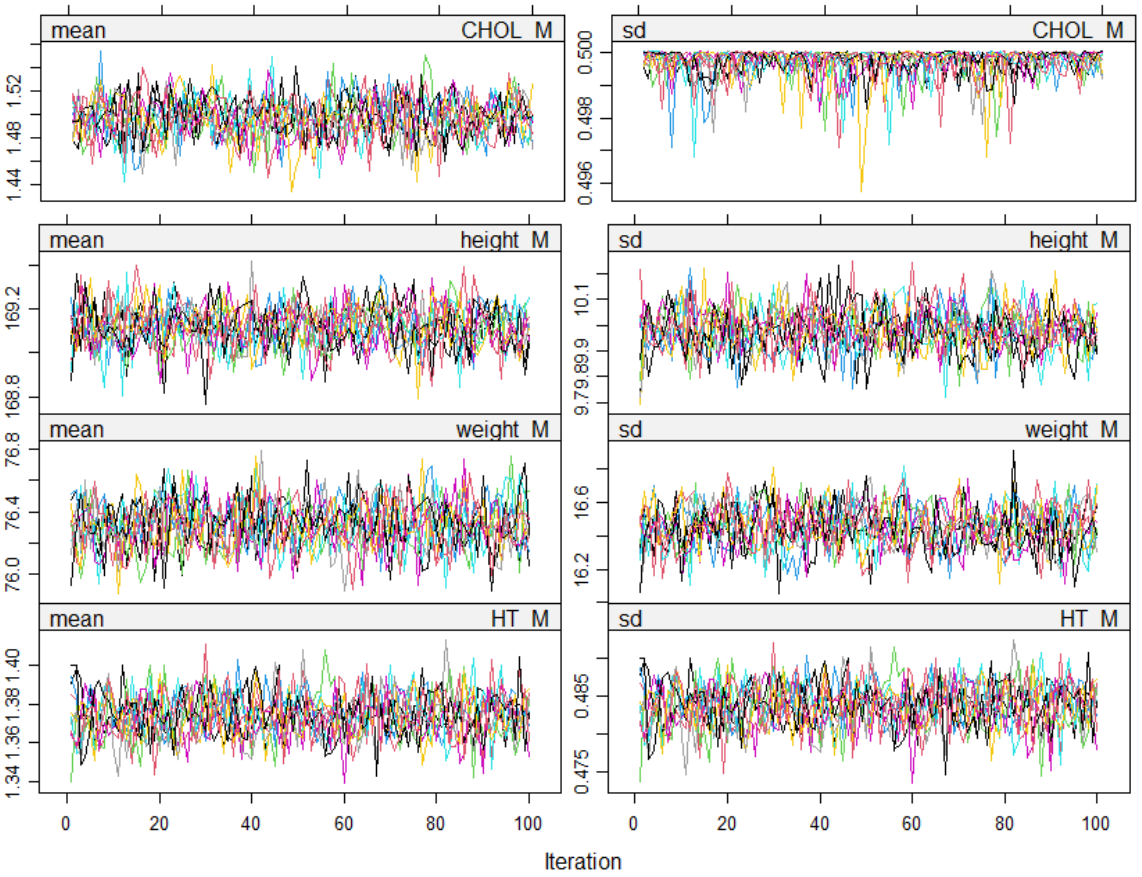

RF MICE

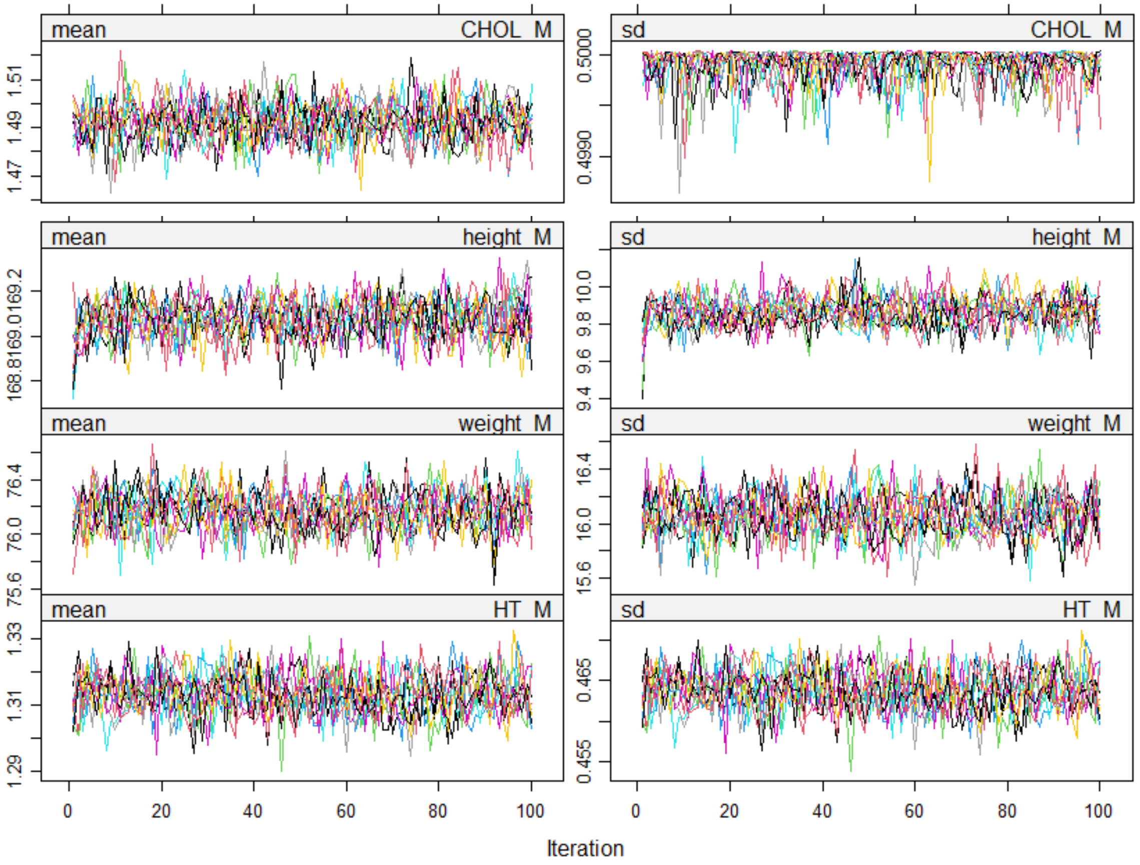

Imputation model includes age, sex, education level, self-reported height, self-reported weight, self-reported hypertension and self-reported hypercholesterolemia. Interactions terms were included in the classic imputation model: between self-reported height and age, and between self-reported hypercholesterolemia and age, education level and sex.
