# Supplementary material for: Using random-forest multiple imputation to address bias of self-reported anthropometric measures, hypertension and hypercholesterolemia in the Belgian health interview survey
Source: BMC Med Res Methodol. 2023 Mar 25;23:69. doi: 10.1186/s12874-023-01892-x (PMC10040120; doi:10.1186/s12874-023-01892-x)
Supplement: Supplementary file 20 — Additional file 20. Prevalence estimates of overweight, obesity, hypertension and hypercholesterolemia in Belgium using self-reported, measured and adjusted BHIS data for 2008, 2013, and 2018. [file 12874_2023_1892_MOESM20_ESM.pdf]

Additional file 20. Prevalence estimates of overweight, obesity, hypertension and hypercholesterolemia in Belgium using self-reported, measured and adjusted BHIS data for 2008, 2013 and 2018.

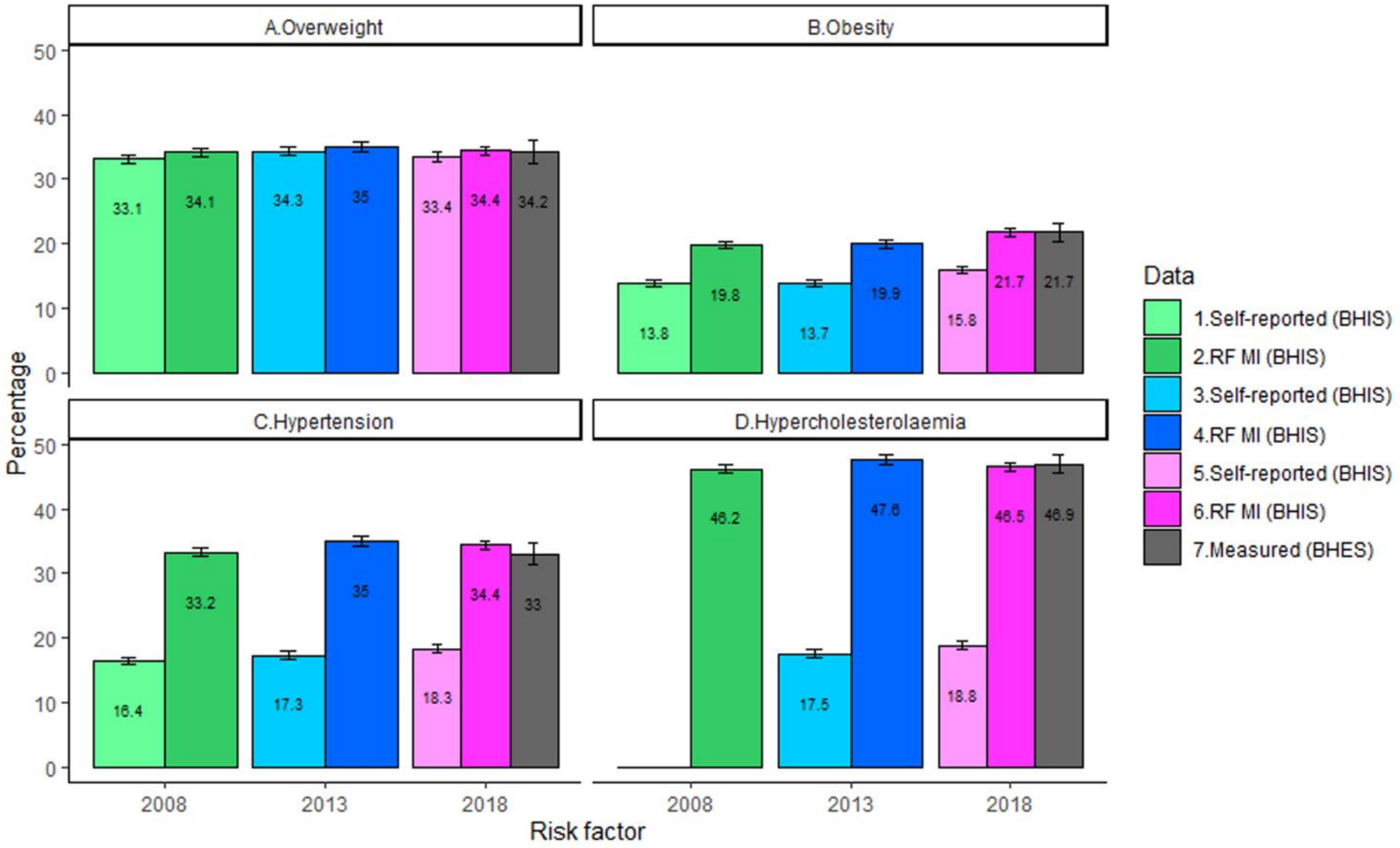


Classic MI: classic multiple imputation. RF MI: random-forest multiple imputation. Regression calibration and multiple imputation model included age, sex, education level and the SR health conditions. Error bars represent one standard deviation of uncertainty of the estimates.
