# Supplementary material for: Using random-forest multiple imputation to address bias of self-reported anthropometric measures, hypertension and hypercholesterolemia in the Belgian health interview survey
Source: BMC Med Res Methodol. 2023 Mar 25;23:69. doi: 10.1186/s12874-023-01892-x (PMC10040120; doi:10.1186/s12874-023-01892-x)
Supplement: Supplementary file 21 — Additional file 21. Ratio of estimated standard errors: BELHES 2018 clinical/adjusted BHIS 2008-2013-2018. [file 12874_2023_1892_MOESM21_ESM.pdf]

Additional file 21. Ratio of estimated standard errors: BELHES 2018 clinical/adjusted BHIS 2008-2013-2018

	RF MI: 2018 BHIS only (n=9439)	RF MI: 2008-2013-2018 BHIS (n=27536)		
	2018	2008	2013	2018
Overweight	1.57	2.62	2.43	2.62
Obesity	1.81	2.64	2.64	2.42
Hypertension	1.79	2.62	2.43	2.43
Hypercholesterolemia	1.21	2.43	2.27	2.07

BHIS: Belgian health interview survey, BELHES: Belgian Health examination survey
